# Dr. John Shires

**Published:** 1912-04-06

**Authors:** 


### Obituary.

Dr. John Shires, M.D., of Shipley, Yorkshire, has died after a long illness in his sixty-seventh year. He was a member of the University of Aberdeen, but his first qualification was taken at Edinburgh in 1868. The greater part of his medical work was done in Liversedge, where he held the office of medical officer of health. He was also appointed honorary surgeon to the Dewsbury Infirmary. Later on he gave up his practice, and studying law at the Middle Temple, he was called to the Bar in the nineties. After a period of travel Dr. Shires took up a practice at Shipley, where he was held in great esteem. He was a Fellow of the Society of Medical Officers of Health, and an examiner to the St. John Ambulance Association.

